# Respiratory morbidity at age 4 across COVID-19 and pre-pandemic birth cohorts: the role of atopic risk

**DOI:** 10.1007/s00431-026-07394-4

**Published:** 2026-10-05

**Authors:** Alma Kamar, Avigdor Mandelberg, NatalyRosenfeld, Diana Tasher, Marina Michleson, Adi Meridor Eisner, Adi Ovadia, Yaara Shen Cohen, Amit Manor, Keren Armoni Domany

**Affiliations:** 1 Pediatric Department, Wolfson Medical Center, Holon, Israel; 2 Pediatric Pulmonology Unit, Wolfson Medical Center, 5822012 Holon, Israel; 3 Gray Faculty of Medical and Health Sciences, Tel Aviv University, Tel Aviv, Israel

**Keywords:** COVID-19 pandemic, Birth cohort, Atopy, Wheezing, Asthma

## Abstract

The COVID-19 pandemic dramatically reduced early-life respiratory viral exposures, yet the long-term respiratory consequences for pandemic-born children remain unclear. We aimed to compare respiratory morbidity at age 4 between children born during versus before the pandemic and assess whether atopic risk modifies this association. This single-center birth cohort follow-up study included children born in February–March 2020 (COVID-19 cohort) and pre-pandemic controls born during the same period in 2019. Respiratory outcomes at age 4 were assessed using a structured telephone questionnaire (The International Study of Wheezing in Infants). The primary outcome was composite respiratory morbidity (CRM), defined as use of a short-acting bronchodilator (SABA), inhaled and/or oral corticosteroids, and/or emergency room visits for respiratory symptoms. Of 588 eligible children, 329 (55.9%) completed follow-up (139 COVID-19, 190 controls); baseline characteristics, including atopy prevalence, were comparable between cohorts. CRM during the fourth year of life was significantly more common in the COVID-19 cohort (39.6% vs. 27.4%, *p* = 0.02). In stratified analyses, CRM was markedly higher among atopic-risk pandemic-born children (atopic subgroup: *n* = 47 COVID-19, *n* = 68 controls) (66% vs. 27.9%, *p* < 0.001), but not among non-atopic children (26.1% vs. 27%). Multivariable logistic regression confirmed a significant COVID-19 cohort × atopy interaction for CRM (OR 5.04, 95% CI 1.83–13.89, *p* = 0.002), while neither factor alone was independently associated with the outcome.

*Conclusions*: Birth during the COVID-19 pandemic was associated with increased preschool respiratory morbidity, particularly among children with atopic risk. These findings suggest that altered early-life microbial exposures may modify respiratory morbidity trajectories according to host atopic susceptibility.

**Table:** 

**What is Known:** • *Children born during the COVID-19 pandemic had fewer respiratory infections and wheezing episodes in infancy due to reduced viral circulation.* • *Atopic predisposition is among the strongest risk factors for recurrent wheezing and persistent asthma in preschool children.*	
**What is New:** • *Pandemic-born children show significantly higher composite respiratory morbidity at age 4, but exclusively among those with atopic risk (OR 5.04).* • *Neither pandemic birth nor atopy alone predicts excess morbidity—only their combination does, suggesting host susceptibility shapes long-term respiratory trajectories.*

**Supplementary Information:**

The online version contains supplementary material available at https://doi.org/10.1007/s00431-026-07394-4.

## Introduction

Respiratory morbidity is among the most common medical conditions of early childhood, affecting up to one-third of children by age 3 and nearly half by early school age, representing a major source of healthcare utilization, parental concern, and long-term disease burden [1, 2]. Respiratory symptoms in young children are frequently driven by viral infections, particularly respiratory syncytial virus (RSV) and rhinovirus, which are strongly linked to recurrent wheezing and the subsequent development of persistent asthma depending on host susceptibility [3–6].

Early childhood is a critical window for immune development, during which exposure to respiratory viruses and environmental microorganisms shapes immune tolerance and inflammatory regulation [7, 8]. The biodiversity hypothesis posits that insufficient microbial stimulation during this period impairs immune regulatory pathways and promotes allergic sensitization, particularly in genetically predisposed children [8]. Atopy, encompassing atopic dermatitis, allergic rhinitis, and food allergy, as well as parental asthma is among the strongest established risk factors for persistent wheezing and childhood asthma [3]. Atopic children may be particularly vulnerable to the respiratory consequences of disrupted early-life microbial exposure [8].

The COVID-19 pandemic provided a unique opportunity to examine the long-term respiratory consequences of markedly reduced early-life viral exposure in a prospectively followed birth cohort. Public health measures including lockdowns, daycare closures, and enhanced hygiene practices led to a dramatic and well-documented decline in circulating respiratory viruses among infants and young children during 2020–2021 [9–11]. Several studies, including our previous report from this birth cohort, demonstrated significantly lower rates of wheezing episodes and respiratory hospitalizations during the first year of life among pandemic-born children compared with pre-pandemic controls [1, 9–11]. These findings raised a critical, unresolved question: does reduced early-life viral exposure confer lasting respiratory benefit, or does it merely delay inevitable morbidity and does the answer depend on the child's underlying susceptibility?

To date, few studies have examined respiratory outcomes in pandemic-born children beyond infancy, with inconsistent findings [12–14]. None extended follow-up beyond 30 months, used composite respiratory outcomes, or examined atopic predisposition as a potential modifier, despite its established role as one of the strongest predictors of persistent wheeze at preschool age [3, 15]. Thus, the current study aimed to compare composite respiratory morbidity at age 4 between children born during the COVID-19 pandemic and a pre-pandemic cohort, and to assess whether atopic risk modifies this relationship.

## Materials and methods

### Study design and setting

This was a prospective single-center birth cohort study conducted at the “Edith Wolfson Medical Center,” a public hospital in Israel. It included infants born between February 1, 2020, and March 31, 2020 (COVID-19 group), and a control group of infants born in the same months in 2019. All participants were part of the original cohort assessed during their first year of life [1]. We excluded children with major genetic syndromes, congenital lung disease, severe immunologic conditions, or significant neurological impairments.

### Data collection and questionnaire

We contacted the parents when the children were 4 and administered a structured telephone questionnaire related to their 4th year of life, based on the International Study of Wheezing in Infants (EISL) [16], a validated instrument for assessing wheezing and respiratory symptoms in early childhood with additional relevant questions related to respiratory morbidity. Outcome definitions were primarily derived from the EISL framework and further informed by the Global Initiative for Asthma (GINA) guidelines (2025) [17].

In addition to the core EISL items, the questionnaire was expanded to capture respiratory outcomes relevant to preschool-aged children, including exercise-induced respiratory symptom exacerbation, physician-diagnosed pneumonia, and hospitalization due to respiratory causes. The questionnaire included items occurred during the previous years: symptom frequency, exertional dyspnea, medication use such as inhaled corticosteroids (ICS), short acting beta agonist (SABA), and systemic corticosteroids, emergency department (ER visits), hospitalizations, and pneumonia diagnosis, as well as allergic comorbidities (e.g., atopic dermatitis, food allergy, allergic rhinitis), family atopy, and environmental exposures (e.g., daycare attendance, passive smoke exposure). Overall, atopic risk was defined as at least one of the following: atopic dermatitis, allergic rhinitis, food allergy, and/or family atopy. “Family atopy” was defined as family history of asthma and/or allergic disease (atopic dermatitis, allergic rhinitis, or food allergy). Data were collected via Microsoft Forms and administered by trained physicians and a pulmonary technician. Informed verbal consent was obtained prior to participation. Atopic dermatitis, allergic rhinitis, food allergy, and family atopic history were ascertained by parental report at the age 4 interview and reflect cumulative status from birth to the time of assessment. The full questionnaire is provided in Supplementary Appendix S1.

The primary outcome CRM was defined as the occurrence of at least one of the following during the fourth year of life: use of SABA, ICS, or oral corticosteroids, and/or at least one emergency room (ER) visit for respiratory symptoms. Children meeting any of these criteria were classified as CRM = 1; all others were classified as CRM = 0. We chose this utilization-based composite, rather than parent-reported wheezing frequency, because preschool wheeze is under-recognized and inconsistently reported by caregivers, whereas medication use and ER visits require an independent clinical encounter and so offer a more objective marker of disease burden [15, 18–20].

Secondary outcomes included respiratory symptoms and clinical events not captured by the CRM outcome, assessed at 4 years of age. These included any wheezing, recurrent wheezing (≥ 2 episodes), exercise-induced respiratory-symptom exacerbation, physician-diagnosed pneumonia, and hospitalization for respiratory causes or respiratory medication use in the past year as mentioned above.

The study was approved by the Helsinki Committee of Wolfson Medical Center (approval number WOMC-0152–23). Verbal informed consent was obtained in accordance with institutional ethics committee approval, which waived written consent given the observational telephone-based design.

### Statistical analysis

Statistical analyses were conducted using SPSS version 31.0. Normality was evaluated using histograms as well as the Shapiro–Wilk test. Continuous variables were compared using independent *t*-tests or Mann–Whitney *U*-tests as appropriate; categorical variables were compared using chi-square or Fisher’s exact test.

Respiratory outcomes were analyzed in three sequential steps. First, to characterize the raw clinical signal, outcomes were compared between cohorts and within cohorts and atopic risk strata (atopic and non-atopic subgroups) using chi-square or Fisher’s exact test. Second, to illustrate the pattern of risk across all four cohort–atopy combinations, unadjusted odds ratios were estimated using a four-level composite variable (Non-atopic/Control, Non-atopic/COVID-19, Atopic/Control, and Atopic/COVID-19), with the Non-atopic/Control group as the reference. This step was intended to characterize the specificity of risk to the Atopic/COVID-19 subgroup and is presented descriptively. Third, and as the primary analytic test of effect modification, multivariable logistic regression models were fitted for outcomes with an events-per-variable ratio ≥ 10. Each model included the main effects of birth cohort and atopy status, their interaction term (cohort × atopy), and the following pre-specified covariates: sex, prematurity, and parental smoking. The interaction term was the primary inferential target; a significant interaction odds ratio indicates that the combined effect of atopic predisposition and pandemic birth exceeds what either factor predicts independently. Outcomes with an events-per-variable (EPV) ratio below this threshold were not modelled. To determine whether the atopy × COVID-19 cohort interaction was attributable to the child’s own atopic phenotype or to familial atopic loading, the primary model was repeated with the atopy exposure restricted to child-only atopy (Model 2) and family-only atopy (Model 3), each adjusted for the same covariates as the primary model. A missing data analysis compared participants who completed follow-up with those lost to follow-up or who declined participation, across key baseline demographic and clinical characteristics. To address potential attrition bias, we performed an inverse-probability-of-participation-weighted sensitivity analysis, using a logistic model of participation (predictors: cohort, ethnicity, sex, gestational age, birth weight, parental smoking, breastfeeding, baseline atopic indicators) to derive stabilized weights, which were applied to the primary CRM model with robust standard errors. All tests were two-sided; *p* < 0.05 was considered statistically significant.

## Results

### Baseline demographic and clinical characteristics

A total of 588 children were initially enrolled in the 1-year follow-up cohort. Of these, 329 children (55.9%) completed the current follow-up assessment at 4 years of age, while 259 (44.1%) were lost to follow-up or refused to participate in the current study. Among participants who completed follow-up, 139 (45%) belonged to the COVID-19 cohort and 190 (55%) to the control cohort. Given the current size of the final analytic cohort, a missing data analysis was performed. Comparison of participants with complete data and those with missing data demonstrated no significant differences in key baseline demographic and clinical characteristics, including sex, prematurity, parental smoking, and study group allocation. A significant difference was observed only in ethnicity, with a higher proportion of Jewish participants among non-participants (92.6% vs. 86.3%, *p* = 0.02; Appendix Table S1A).

Demographics, atopic, and environmental factors are presented in Tables 1 and 2. Baseline demographic and perinatal characteristics were similar between cohorts, as were rates of atopic dermatitis, food allergy, and allergic rhinitis. Similarly, no differences between groups were found in family history of atopic risk, household smoking exposure, breastfeeding ≥ 3 months.

**Table 1:** Baseline demographic and perinatal characteristics

Characteristic	COVID-19 cohort *N* = 139	Control cohort *N* = 190	*p*-value
Male sex, *n* (%)	75 (54%)	90 (47%)	0.27
Ethnicity (Jewish)	128 (92%)	171 (90%)	0.81
Gestational age < 37wk, *n* (%)	14 (10.1%)	14 (7.4%)	0.43
Week of gestational age	38.79 ± 1.98	38.9 ± 1.93	0.35
Birth weight (g)	3225 ± 545	3222 ± 524	0.96
Cesarean delivery (%)	18 (12.9%)	13 (6.8%)	0.08

**Table 2:** Atopy and environmental characteristics

Characteristic	COVID-19 cohort *N* = 139	Control cohort *N* = 190	*p*-value
Atopic dermatitis, *n* (%)	18 (12.9%)	14 (7.5%)	0.13
Food allergy, *n* (%)	2 (1.4%)	4 (2.1%)	1
Allergic rhinitis, *n* (%)	17 (12.2%)	18 (9.5%)	0.42
Child atopy	38 (27.3%)	49 (25.8%)	0.8
Family atopy^^^, *n* (%)	30 (21.6%)	50 (26.5%)	0.36
Overall atopy risk^#^	47 (33.8%)	68 (35.8%)	0.73
Parental smoking, *n* (%)	53 (38.1%)	78 (41.1%)	0.65
Breastfeeding ≥ 3 months* n*	71 (51.1%)	109 (57.4%)	0.27

^^^Family atopy including family history of either asthma, atopic dermatitis, or other allergic disease. ^#^At least 1 of the above: atopic dermatitis, allergic rhinitis, food allergy, or family atopy

### Clinical outcomes

Respiratory morbidity outcomes at age 4 are detailed in Table 3. The primary composite outcome (CRM) was significantly more common in the COVID-19 cohort compared with controls (39.6% vs. 27.4%, *p* = 0.02). Among secondary outcomes, exercise-induced respiratory symptoms were more frequent in the COVID-19 cohort (15.1% vs. 7.5%, *p* = 0.03). SABA use was numerically higher in the COVID-19 cohort (33.1% vs. 23.7%) but did not reach statistical significance (*p* = 0.06). No significant differences were observed in wheezing prevalence, ICS or OCS use, emergency room visits, hospitalizations, or pneumonia rates. Stratified analyses suggested that the overall cohort difference in CRM was driven by atopic children, as detailed below.

**Table 3:** Respiratory outcomes during 4^th^ year of life

Outcome	COVID-19 cohort *N* = 139	Control cohort *N* = 190	*p*-value
CRM, *n* (%)	55 (39.6%)	52 (27.4%)	**0.02**
Any wheezing, *n* (%)	24 (17.3%)	27 (14.2%)	0.54
Recurrent wheezing (≥ 2), *n* (%)	20 (14.4%)	26 (13.2%)	0.74
SABA use, *n* (%)	46 (33.1%)	45 (23.7%)	**0.06**
ICS use, *n* (%)	39 (28.1%)	41 (21.6%)	0.19
OCS use, *n* (%)	10 (7.2%)	13 (6.8%)	1
ER visit, *n* (%)	10 (7.2%)	12 (6.3%)	0.83
Pneumonia diagnosis, *n* (%)	10 (7.2%)	6 (3.2%)	0.12
Exercise-induced symptoms, *n* (%)	21 (15.1%)	14 (7.5%)	**0.03**

*CRM* composite respiratory morbidity, *ER* emergency room, *ICS* inhaled corticosteroids, *OCS* oral corticosteroids, *SABA* short acting beta agonist

### Atopic risk as a modifier of cohort differences in respiratory morbidity

#### Subgroup analysis by atopic risk

**Fig. 1:**
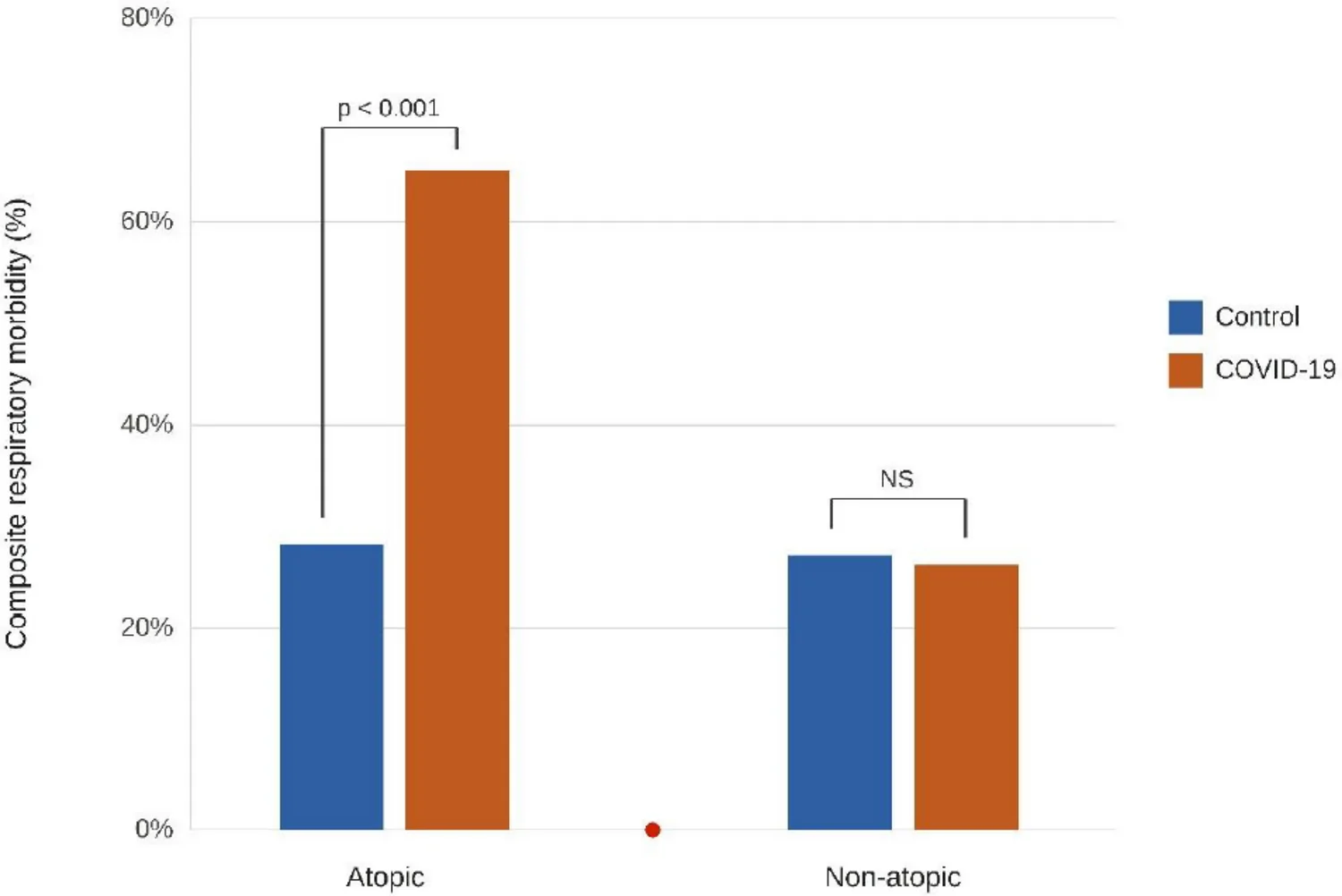
Prevalence of composite respiratory morbidity at age 4 stratified by atopic predisposition and birth cohort

 To explore whether the overall cohort difference in CRM was modified by atopic risk, outcomes were compared separately within atopic and non-atopic children. Among atopic children (atopic subgroup: *n* = 47 COVID-19, *n* = 68 controls), CRM was markedly higher in the COVID-19 cohort compared with controls (66.0% vs. 27.9%, *p* < 0.001), as were SABA use (53.2% vs. 23.5%, *p* = 0.001), ICS use (44.7% vs. 22.1%, *p* = 0.01), and exercise-induced respiratory symptoms (25.5% vs. 10.4%, *p* = 0.04). In contrast, among non-atopic children, no significant differences between cohorts were observed for any outcome, including CRM (26.1% vs. 27.0%), SABA use (22.8% vs. 23.8%), or ICS use (19.6% vs. 21.3%). All other outcomes, including wheezing measures, OCS use, ER visits, and nocturnal symptoms did not differ significantly between cohorts within either atopy stratum (Fig. 1).

#### Unadjusted logistic regression analysis across four cohort–atopy combinations

**Fig. 2:**
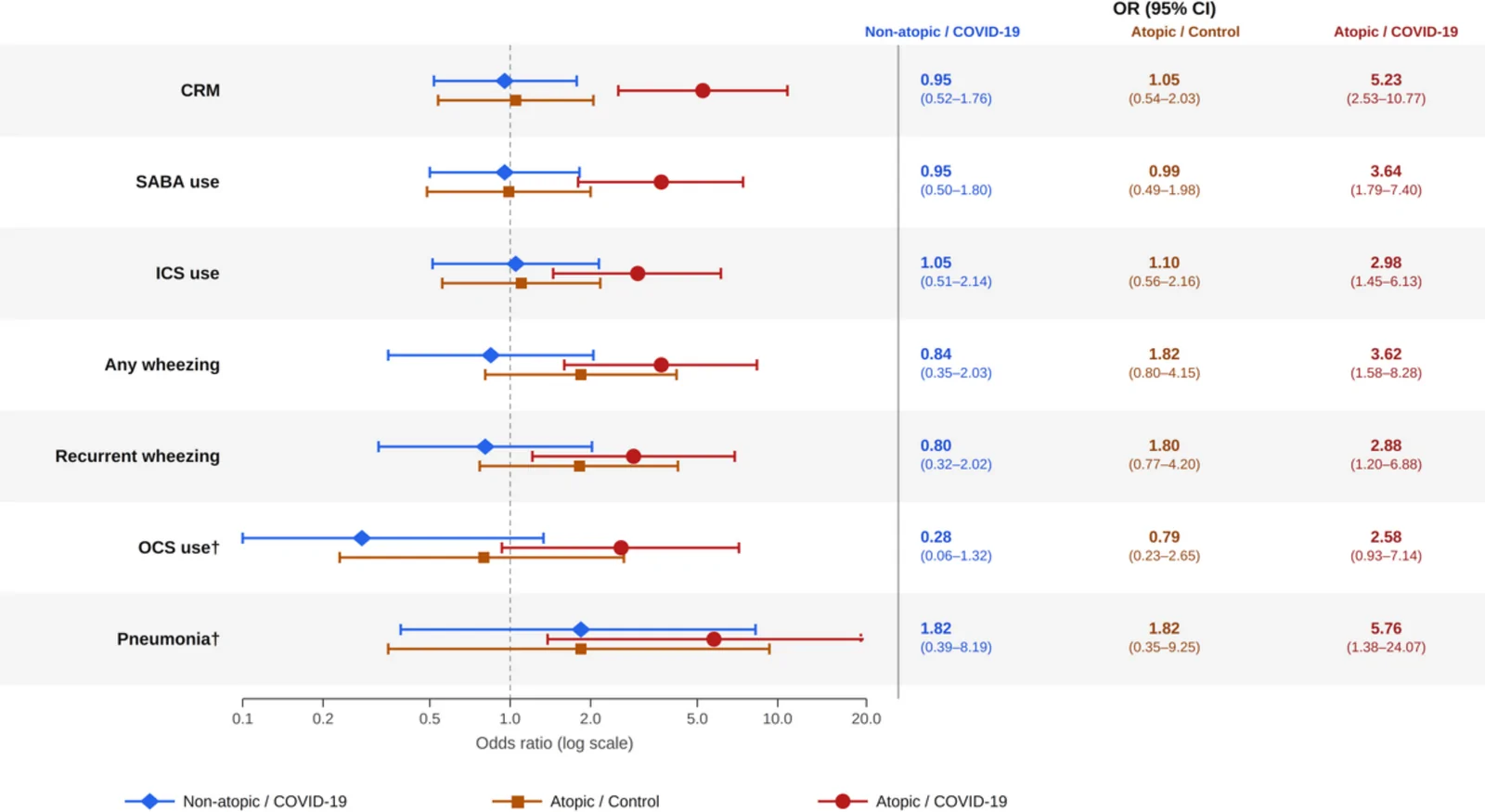
Unadjusted odds ratios for respiratory outcomes at age 4 by cohort–atopy group, relative to the Non-atopic/Control reference group–exploratory analysis. Reference group: Non-atopic/Control. Unadjusted logistic regression. Dashed line at OR = 1. ^†^Interpret with caution: fewer than 25 events. Horizontal bars represent 95% confidence intervals. CRM, composite respiratory morbidity; ICS, inhaled corticosteroids; OCS, oral corticosteroids; SABA, short acting beta agonist

 To characterize the specificity of risk across all four cohort–atopy combinations, unadjusted ORs were estimated descriptively using the composite four-level variable. This confirmed that elevated risk was confined to the Atopic/COVID-19 subgroup, while the Non-atopic/COVID-19 and Atopic/Control groups both showed ORs close to unity across all outcomes. OCS use and pneumonia should be interpreted with caution given fewer than 25 events in these outcomes (Fig. 2).

#### Multivariable logistic regression analysis

 To formally test effect modification, multivariable logistic regression models including cohort, atopy, their interaction term, and pre-specified covariates were fitted for outcomes with events-per-variable ≥ 10. A significant cohort × atopy interaction was confirmed for CRM (adjusted OR 5.04, 95% CI 1.83–13.89, *p* = 0.002), SABA use (adjusted OR 3.73, 95% CI 1.33–10.46, *p* = 0.01), and ICS use (adjusted OR 3.07, 95% CI 1.06–8.84, *p* = 0.04). In each model, neither birth cohort alone nor atopy alone was independently associated with the outcome, confirming that excess respiratory morbidity emerged specifically from their combination (Table 4). Taken together, these findings indicate that the overall elevation in CRM observed in the COVID-19 cohort was driven entirely by atopic children, with no excess morbidity observed among non-atopic pandemic-born children. We then verified that this finding was not attributable to the ethnicity difference between participants and those lost to follow-up. An inverse-probability-of-participation-weighted sensitivity analysis yielded a similar, though somewhat attenuated, cohort × atopy interaction for CRM (weighted OR 4.02, 95% CI 1.38–11.76, *p* = 0.01).

**Table 4:** Multivariable logistic regression of respiratory outcomes by atopy-cohort combined groups adjusted for sex, prematurity and parental smoking

Outcome	Overall Atopy OR (95% CI)	COVID-19 cohort OR (95% CI)	Overall atopy × COVID-19 interaction OR (95% CI)	Adj-*p*- interaction
***Model 1***				
CRM	1.09 (0.56–2.12)	0.96 (0.52–1.77)	**5.04 (1.83–13.89)**	**0.002**
SABA use	1.02 (0.50–2.05)	0.96 (0.50–1.82)	**3.73 (1.33–10.46)**	**0.012**
ICS use	1.08 (0.52–2.22)	0.90 (0.46–1.77)	**3.07 (1.06–8.84)**	**0.038**

All models adjusted for parental smoking, sex, and prematurity. Neither main effect nor any covariate reached statistical significance in any model. Outcomes with events-per-variable < 10 were not modeled and are reported descriptively only. *CRM* composite respiratory morbidity, *ICS* inhaled corticosteroids, *SABA* short acting beta agonist

To determine whether the interaction was driven by the child’s or the family atopy, we repeated this model restricting exposure to child-only and family-only atopy (Table 5). The interaction was not significant for child atopy (CRM = OR 1.8, ICS = OR 1.83, SABA = OR 1.71; all adj-*p* ≥ 0.28), but showed a pattern consistent with the primary model for family atopy, reaching significance for CRM (OR 4.5, 95% CI 1.49–13.98, adj-*p* = 0.01). This suggests the overall atopy × COVID-19 cohort interaction is driven predominantly by family atopy rather than child atopy.

**Table 5:** Sensitivity analysis decomposing the atopy × COVID-19 cohort interaction by source of atopy: Child-Only versus Family atopy

Outcome Model 2	Atopic child OR (95% CI)	COVID-19 cohort OR (95% CI)	Atopic child × COVID-19 interaction OR (95% CI)	Adj-*p*- interaction
CRM	2.15 (1.06–4.3)*	1.46 (0.8–2.57)	1.8 (0.62–5.25)	0.28
SABA use	1.912 (0.092–3.97)	3.33 (0.72–2.45)	1.71 (0.58–5.07)	0.33
ICS use	2.27 (1.08–4.79)	1.12 (0.58–2.17)	1.83 (0.60–5.56)	0.28
*Model 3*	Family atopy OR (95% CI)	COVID-19 cohort OR (95% CI)	Family atopy × COVID-19 interaction OR (95% CI)	Adj-*p*- interaction
CRM	1.2 (0.69–2.08)	0.92 (0.44–1.92)	4.5 (1.49–13.98)	0.01
SABA use	1.22 (0.687–2.17)	1.019 (0.475–2.18)	2.87 (0.929–8.8)	0.07
ICS use	1.045 (0.476–2.296)	1.13 (0.623–2.064)	2.29 (0.721–7.28)	0.16

Models 2 and 3 adjusted for parental smoking, sex, and prematurity. *CRM* composite respiratory morbidity, *ICS* inhaled corticosteroids, *SABA* short acting beta agonist. *p < 0.05

## Discussion

In this prospective birth cohort study, children born during the COVID-19 pandemic exhibited significantly higher CRM reflecting clinical and healthcare utilization burden, at age 4 compared with pre-pandemic controls. Multivariable analysis revealed a significant interaction between atopic predisposition and COVID-19-birth cohort: neither factor independently associated with CRM, SABA use, or ICS use, yet their combination was associated with markedly elevated risk. These findings suggest that the early-life respiratory protection observed during the pandemic did not translate into lasting benefit at preschool age, and that the long-term consequences of altered early-life exposures may be associated with underlying atopy-host susceptibility. However, no differences were found for most of our secondary outcomes. In particular, “any wheezing” and “recurrent wheezing” did not differ significantly between cohorts, while medication use and utilization-based outcomes did; this dissociation suggests that CRM captures increased treatment intensity or a lower threshold for pharmacologic intervention among atopic COVID-19-cohort children, rather than a higher raw frequency of wheezing episodes, and is therefore better interpreted as a composite marker of clinical/healthcare burden than as a direct proxy for underlying symptom severity.

In our previous report from this cohort, pandemic-born children experienced significantly fewer wheezing episodes and respiratory hospitalizations during the first year of life [1], consistent with the well-documented suppression of respiratory viral circulation during lockdown periods [9–11]. The current follow-up at age 4 presents a different picture: not only has the early protection disappeared, but atopic children in the pandemic cohort demonstrate substantially higher respiratory morbidity than their pre-pandemic counterparts. This pattern of early benefit followed by apparent excess morbidity in susceptible individuals raises the possibility that reduced early-life infectious exposure may defer rather than eliminate the clinical expression of respiratory disease, at least in children with atopic risk.

Several studies have examined respiratory outcomes in pandemic-born children beyond infancy, though most are limited to the first 18–30 months of life and rely on administrative data. Barbieri et al. reported a substantial reduction in wheezing episodes and less use of respiratory medicines up to 30 months among lockdown-born children in Italy [12]. Kelderer et al. similarly found reduced respiratory infections and antibiotic use up to 18 months in a Swedish population-based cohort, while noting that the longer-term effect on asthma risk remained unknown [14]. A nationwide Israeli study using Clalit Health Services data identified that reduced early-life exposure to RSV during the COVID-19 pandemic did not reduce later asthma-related healthcare use at age 1–3 years [13]. Lachover-Roth et al., using an Israeli prospective birth cohort followed to 12 months, found that infants born after lockdown restrictions were eased showed significantly higher rates of airway hyperresponsiveness compared with those born before or during the pandemic, with no significant differences in other atopic comorbidities—a pattern that may represent the early respiratory correlate of the morbidity we observed at preschool age in atopic children [21]. Our findings extend follow-up to age 4 and suggest that cohort differences in respiratory morbidity may emerge or intensify at preschool age, particularly among atopic children, a pattern not captured in earlier and/or registry-based studies.

The methodological differences between our study and prior work merit consideration. Administrative registry studies capture medication dispensing and healthcare contacts but may not fully assess symptom burden or exercise limitation, both of which were included in our structured questionnaire. Age 4 may represent a relevant follow-up point, as atopic predisposition appears to become an increasingly reliable predictor of ongoing respiratory morbidity during the preschool years [22].

The biological basis for the observed atopy–cohort interaction is consistent with current understanding of early immune development. The first years of life represent a critical window during which microbial and viral exposures shape immune regulatory pathways and promote tolerance [7, 8]. The biodiversity hypothesis proposes that insufficient early microbial stimulation may impair these processes and promote allergic sensitization, particularly in genetically predisposed individuals [3, 8], while the related immune debt hypothesis holds that reduced early-life exposure may defer rather than eliminate respiratory morbidity [23]. Pandemic-related reductions in early viral exposure may have selectively altered immune maturation in children with an underlying atopic predisposition, resulting in amplified respiratory morbidity when viral exposure resumed, an effect that, in our cohort, appeared confined to atopic children, with no cohort differences observed among non-atopic children across any outcomes.

Two alternative explanations merit consideration, though neither is fully testable with our data. First, the same biodiversity mechanism could run in the opposite causal direction: reduced early-life infections during the pandemic may themselves have promoted allergic sensitization, making atopy partly a consequence of pandemic-era birth rather than only a pre-existing modifier that filtered its effects. This is consistent with the non-significant trend toward higher atopic dermatitis and allergic rhinitis in the COVID-19 cohort (Table 2). Because atopy was ascertained cross-sectionally at age 4 rather than independently of pandemic exposure, our data cannot formally distinguish a pre-existing modifier from a pandemic-induced one. Second, the atopy–CRM association could simply reflect ordinary atopic march progression rather than a pandemic-specific effect, since atopy is a well-established predictor of later wheeze and rescue-medication use independent of pandemic exposure. This alone, however, would not explain why the association was more pronounced in the pandemic-born cohort than among atopic children in the control group, pointing to a cohort-specific effect beyond ordinary march progression, though an accelerated-trajectory variant cannot be fully excluded given the available data.

It should be noted that when atopic risk was decomposed into child atopy and family atopy, the cohort interaction was significant only for family atopy, not for child atopy, suggesting that family history, rather than child atopy, may be the more relevant modifier of the observed association. This null finding for child atopy should, however, be interpreted with caution: it was ascertained at age 4, and conditions such as allergic rhinitis often emerge later in childhood [24, 25], so some children classified as non-atopic may not yet have manifested atopic disease—potentially biasing this interaction toward the null. This maturation lag does not apply to family atopy, which may partly explain its comparatively stronger signal. Given the smaller strata in this post-hoc analysis, these findings remain hypothesis-generating and warrant confirmation in a larger sample.

These findings have potential clinical implications. Atopic predisposition may be an important modifier of respiratory outcomes in children born during periods of reduced infectious burden, and this susceptibility should be considered in clinical follow-up. As discussed above, these findings also support the use of composite respiratory outcomes over wheezing alone when assessing preschool respiratory health [15, 18–20]. Whether similar patterns will be observed in the context of reduced early RSV exposure following the introduction of nirsevimab or maternal RSV vaccination is an important question that warrants prospective evaluation.

The strengths of this study include its prospective design, availability of detailed early-life atopy and environmental exposure data, use of a structured questionnaire at a clinically relevant follow-up age, and comparison with a well-characterized pre-pandemic control cohort from the same institution.

Several limitations should be acknowledged. Approximately 44% of the original cohort was lost to follow-up, raising the possibility of attrition bias. Loss to follow-up was balanced between study-arm allocations (*p* = 0.86) and across all other baseline variables listed in Table S1, except ethnicity, which was more prevalent among non-participants. To address this, we performed an inverse-probability-of-participation-weighted sensitivity analysis; the cohort–atopy interaction remained significant after reweighting (weighted OR 4.02 vs. 5.04, unweighted), indicating that this imbalance is unlikely to fully explain the observed association, though residual confounding from unmeasured factors cannot be excluded. Secondly, respiratory outcomes were parent-reported and are therefore subject to recall bias and potential misclassification. Specifically, atopic exposures and outcomes were both reported by the same parent at a single age-4 interview, raising the possibility of shared-informant bias: parents of more symptomatic children may have been more likely to recall and report co-occurring atopic diagnoses, potentially inflating the observed association independent of a true biological effect. Furthermore, objective measures of lung function, atopic sensitization, and immune markers were not available, limiting mechanistic interpretation. The timing and microbiological confirmation of respiratory infections after lockdown were not captured, precluding direct testing of the immune debt hypothesis. Furthermore, a temporal asymmetry exists between cohorts: controls (born 2019) were assessed at age 4 in 2023, while pandemic-born children (born 2020) were assessed in 2024. Both assessments occurred after the end of pandemic restrictions and resumption of normal viral circulation, making a simple calendar-year bias unlikely. We also cannot exclude a priori a secular shift in prescribing or health-seeking behavior between the two years; however, this is an unlikely explanation, as excess morbidity was largely confined to atopic children within the 2024 cohort, while non-atopic children assessed in the same year resembled 2023 controls—a pattern a general secular shift would not readily produce. GINA criteria for preschool wheeze/asthma were also relatively similar across the two time periods*.* Finally, as a single-center study with limited sample size, generalizability to other populations and healthcare settings may be limited.

In conclusion, children born during the COVID-19 pandemic showed increased composite respiratory morbidity reflecting clinical and healthcare utilization burden at preschool age compared with pre-pandemic controls, driven specifically by atopic predisposed children. These findings suggest that altered early-life microbial exposures may reshape rather than eliminate respiratory morbidity trajectories, with long-term outcomes appearing to depend substantially on underlying host susceptibility, such as atopy. Continued follow-up into school age will be important to determine whether these trajectories persist and contribute to the future burden of childhood respiratory morbidity and asthma.

## Supplementary Information

Below is the link to the electronic supplementary material.

ESM 1 DOCX (19.9 KB)

ESM 2 DOCX (15.1 KB)

## Data Availability

All data supporting the findings of this study are available within the paper and its Supplementary Information. The raw data will be available upon reasonable request for clinical and research purposes.

## Abbreviations

CRM: Composite respiratory morbidity
EISL: International Study of Wheezing in Infants
GINA: Global Initiative for Asthma
ER: Emergency department
ICS: Inhaled corticosteroids
OR: Odds ratio
RSV: Respiratory syncytial virus
SABA: Short acting beta agonist
