# Supplementary material for: Respiratory morbidity at age 4 across COVID-19 and pre-pandemic birth cohorts: the role of atopic risk
Source: Eur J Pediatr. 2026 Oct 5;185(11):802. doi: 10.1007/s00431-026-07394-4 (PMC13635026; doi:10.1007/s00431-026-07394-4)
Supplement: Supplementary file 1 [file 431_2026_7394_MOESM1_ESM.docx]

**Supplementary Appendix S1. Structured Questionnaire**

**Respiratory symptoms and healthcare utilization**

**1. How many episodes of wheezing did your child experience during the past year?**

- 0
- 1
- 2
- 3
- ≥4

**2. During the past year, has your child experienced worsening of respiratory symptoms at night?**

- Yes
- No

**3. During the past year, has your child experienced shortness of breath, wheezing, or cough during physical activity, laughing, exposure to animals, or exposure to smoke?**

- Yes
- No

**4. During the past year, has your child become easily fatigued during physical activity, including walking?**

- Yes
- No

**5. During the past year, has your child experienced wheezing, shortness of breath, or cough lasting more than 10 days during a viral illness?**

- Yes
- No

**6. How many times during the past year did your child use inhaled short-acting bronchodilators e.g. salbutamol (Ventolin) or terbutaline (Bricanyl)?**

- 0
- 1–2
- 3–4
- ≥5

**7. How many times during the past year was your child treated with inhaled corticosteroids e.g. fluticasone (Flixotide), budesonide (Budicort)?**

- 0
- 1–2
- 3–4
- Regular/continuous treatment

**8. How many times during the past year was your child treated with systemic corticosteroids (e.g., oral corticosteroids such as Betnesol)?**

- 0
- 1–2
- 3–4
- ≥5

**9. How many times during the past year did your child require medical evaluation in an emergency department, urgent care clinic, or other acute medical care center because of a respiratory illness?**

- 0
- 1
- 2
- 3
- ≥4

**10. How many times was your child hospitalized during the past year because of a respiratory illness?**

- 0
- 1
- 2
- 3
- ≥4

**11. Has your child had pneumonia during the past year?**

- Yes
- No

**12. Is your child currently under the care of a pulmonologist?**

- Yes
- No

**Atopic and family history**

**13. Does your child have atopic dermatitis ?**

- Yes
- No

**14. Does your child have a food allergy?**

- Yes
- No

**15. Is there a family history of atopy (asthma, food allergy, atopic dermatitis or allergic rhinitis)?**

- Yes
- No

**16. Does your child have chronic or allergic rhinitis?**

- Yes
- No

**Other exposures and medical history**

**17. At what age did your child start attending daycare?**

- Before 1 year of age
- 1 year of age or older
- 2 years of age or older
- 3 years of age or older

**18. Has your child had COVID-19 during the past year?**

- Yes
- No

**Table S1. Baseline characteristics of participants and non-participants**

| **Variable** | **Participants (n=329)** | **Non-participants (n=259)** | **p-value** |
| --- | --- | --- | --- |
| *Male sex, n (%)* | 164 (47.7%) | 124 (49.2%) | 0.738 |
| *Jewish ethnicity n (%)* | 297 (86.3%) | 226 (92.6%) | **0.021** |
| *Preterm Birth n (%)* | 35 (10.2%) | 22 (9%) | 0.7 |
| *Smoking parents n (%)* | 143 (42.4%) | 102 (41.8%) | 0.87 |
| *Study group (COVID19 group), n (%)* | 139 (42.2%) | 121 (49.6%) | 0.86 |
| *Parental overall atopy* | 87 (25.8%) | 69 (28.3%) | 0.507 |
| *Food allergy at age 1 year* | 20 (5.8%) | 8 (3.3%) | 0.175 |
| *Atopic dermatitis at age 1 year* | 27 (7.8%) | 28 (11.5%) | 0.151 |
| ***Respiratory morbidity at 1 year of age*** | | | |
| *Wheezing and/or asthma medications* | 99 (40.6%) | 117 (34%) | 0.118 |
