# Supplementary material for: Respiratory morbidity at age 4 across COVID-19 and pre-pandemic birth cohorts: the role of atopic risk
Source: Eur J Pediatr. 2026 Oct 5;185(11):802. doi: 10.1007/s00431-026-07394-4 (PMC13635026; doi:10.1007/s00431-026-07394-4)
Supplement: Supplementary file 2 [file 431_2026_7394_MOESM2_ESM.docx]

| **Variable** | **Participants (n=329)** | **Non-participants (n=259)** | **p-value** |
| --- | --- | --- | --- |
| *Male sex, n (%)* | 164 (47.7%) | 124 (49.2%) | 0.738 |
| *Jewish ethnicity n (%)* | 297 (86.3%) | 226 (92.6%) | **0.021** |
| *Preterm Birth n (%)* | 35 (10.2%) | 22 (9%) | 0.7 |
| *Smoking parents n (%)* | 143 (42.4%) | 102 (41.8%) | 0.87 |
| *Study group (COVID19 group), n (%)* | 139 (42.2%) | 121 (49.6%) | 0.86 |
| *Parental overall atopy* | 87 (25.8%) | 69 (28.3%) | 0.507 |
| *Food allergy at age 1 year* | 20 (5.8%) | 8 (3.3%) | 0.175 |
| *Atopic dermatitis at age 1 year* | 27 (7.8%) | 28 (11.5%) | 0.151 |
| ***Respiratory morbidity at 1 year of age*** | | | |
| *Wheezing and/or asthma medications* | 99 (40.6%) | 117 (34%) | 0.118 |

**Table S1. Baseline characteristics of participants and non-participants**
